# Supplementary material for: Disapproval from romantic partners, friends and parents: Source of criticism regulates prefrontal cortex activity
Source: PLoS One. 2020 Oct 2;15(10):e0229316. doi: 10.1371/journal.pone.0229316 (PMC7531840; doi:10.1371/journal.pone.0229316)
Supplement: Attachment — Submitted filename: 3Crit_Response to Reviewers3.docx [file pone.0229316.s002.docx]

Dear Reviewers,

Thank you for your comments. Please find our response and revisions made to the manuscript based on your comments as highlighted in blue below.

Reviewer #1: Most of my comments have been sufficiently addressed. Below I formulated some remaining issues, most in relation to the processes that the authors describe throughout the manuscript (i.e. self-experienced criticism) compared to what the authors actually investigate (i.e. observed criticism):

Abstract
1. Only in the last three lines of the abstract it becomes clear that the authors actually investigated observed/third-party criticism, instead of self-experienced criticism. This should be clarified from the beginning.

Thank you for pointing this out. We have included a clarification in the beginning of the abstract as follows: “The present study aims to investigate neural responses to observing criticism occurring in the context of originating from three different relationship types: romantic partners, friends, and parents – from a third-party perspective.”

Introduction
1. Although the authors have done a good job in expanding the introduction to include
literature on neural correlates of emotion reaction and observed criticism, the introduction still largely describes processes involved in personal experiences of criticism, which is not what the authors actually investigated. For example, the expectations of high vs low PC individuals seem to be formulated for self-experienced criticism, not observed criticism. It should be made clearer what process the authors actually investigated.

Thank you for your comment on the revisions to the Introduction. We have further clarified on this by specifying in the Introduction as follows:

“Given involvement of these parts of the PFC in emotion reactivity and regulation, coupled with previous findings of decreased prefrontal control to criticism, the present study sought to investigate the neural correlates of responses to other referential criticism specifically in the PFC using functional near-infrared spectroscopy (fNIRS).”

“Hence taking together differences in attributions, levels of perceived face threat and qualities of interpersonal relationships, we expect that individuals differing in PC may also have different responses to other referential criticism originating from different sources.”

“As mentioned, majority of neuroimaging studies discussed above were investigating self-referential criticism with few studies looking at the neural correlates of other referential criticism. In addition, the studies on other referential criticism tended to look primarily at the mPFC as a region of interest and varied in terms of the identity of the “other” as the source of the other referential criticism. In the present study, we aim to investigate PFC activation during exposure to other referential criticism involving an “other” from three different relationship types – romantic partners, friends and parents.

Hence, the present study aims to contribute to existing literature by providing both novel fNIRS data of PFC activation during exposure to other referential criticism and a functional characterization of the mental processes underlying the neural response to criticism observed in the social interactions of others from a third-party perspective while comparing romantic partners, friends, and parents as sources of criticism.”

1. I like how the authors have linked the findings from their study to previous work in the discussion. However, this would be good to also mention in the introduction, as to explain why it is interested to study (neural correlates of) observed criticism in the first place.

Thank you for your comment on the revisions to the Discussion. We have included mention of this in the Introduction as follows: “As mentioned, majority of neuroimaging studies discussed above were investigating self-referential criticism with few studies looking at the neural correlates of other referential criticism. In addition, the studies on other referential criticism tended to look primarily at the mPFC as a region of interest and varied in terms of the identity of the “other” as the source of the other referential criticism. In the present study, we aim to investigate PFC activation during exposure to other referential criticism involving an “other” from three different relationship types – romantic partners, friends and parents.

Hence, the present study aims to contribute to existing literature by providing both novel fNIRS data of PFC activation during exposure to other referential criticism and a functional characterization of the mental processes underlying the neural response to criticism observed in the social interactions of others from a third-party perspective while comparing romantic partners, friends, and parents as sources of criticism.”

Results
2. Following comments in the earlier review, the authors have done a good job in adapting the results section to “In examining the interaction between relationship type and PC rating of mothers, the Pearson product-moment correlations were calculated between beta coefficients for each relationship type and PC rating of mothers (Table 3). The correlation coefficients are as follows: (i) Romantic Partner-PC (Mother) (RPM) was (r = 0.20, n = 44), (ii) Friend-PC (Mother) (FPM) was (r = -0.44, n = 43), and (iii) Parents-PC (Mother) (PPM) was (r = 0.26, n = 41). To examine the significance of the difference between the correlation coefficients, Fisher r-to-z transformation was applied in order to compare the z scores (Table 3). The results summarised in Table 3 showed significantly different correlations for (i) the Romantic Partner-PC (Mother) and Friend-PC (Mother) correlations (Z = 3.02, p < 0.05) and (ii) Friend-PC (Mother) and Parents-PC (Z = -3.25, p < 0.05). The Romantic Partner-PC and Parents-PC correlations were not significantly different (Z = -0.29, p = 0.77 > 0.05). From Fig 5, it can be observed that as PC ratings for the individual’s mother increased, activation of the left middle frontal gyrus of the dlPFC (BA46L) increased when reading the vignettes describing criticism from romantic partners and parents but decreased when reading the vignettes describing criticism from friends.”

Although this adaptation makes this specific section easier to understand it might still be helpful if short interpretations would be provided. For example, the results in Table 3 show significant different correlations for several relationships; how should these findings be interpreted, what do they actually mean for your study?

We have included a short interpretation as follows: “This significant result suggests that PC ratings moderate neural response to criticism originating from different sources of different relationship types, resulting in different activation patterns observed between high and low PC individuals for criticism from the different relationship types.”

Reviewer #3: The present manuscript investigates using functional Near Infrared Spectroscopy (fNIRS) prefrontal (PFC) responses of 49 participants to perceptual criticism from romantic partners, friends and parents (mother and father). The authors reveal an enhancement of the left dorsolateral PFC (DLPFC) links to criticism from romantic partners. At the opposite, a decrease of the left DLPFC is observed for criticism from friends.

The authors already assessed several concerns of reviewer 1. However, I have still important recommendations regarding the methodology.

Major concerns

1. The authors described in the introduction the importance of psychiatric disorders on perceptual criticism. Yet, they did not exclude participants with such disorders in the present study. Even if the authors considered this point in the discussion, as a limitation, I think it is a real issue here. If few participants had psychiatric or neurological disorders, the results observed could be totally biased. I strongly recommend to the authors to contact a posteriori the 49 participants to ensure that all are healthy. If not, the authors should exclude the "non-healthy" participants from the analysis.

We acknowledge that we were not clear in our previous revision – while the participants were not screened as part of our experimental procedure, the participants were recruited from a research participant pool. The research participant pool itself has an exclusion criteria – participants with psychiatric disorders are excluded.

We have clarified this accordingly in the Recruitment subsection as follows: “Having a psychiatric disorder was an exclusion criteria for participants.“ Similarly, we have also clarified in the Limitations regarding future studies as follows: “Although the present study was conducted only on healthy individuals, future studies can look to investigate whether there are differences between individuals diagnosed with psychiatric disorders, such as mood disorders and social anxiety, and healthy individuals in terms of processing criticism observed in the social interactions of others.”

2. The experimental paradigm only included one trial per condition. Even if each trial was presented during 90 seconds, the data acquisition was quiet low for a fNIRS study (+/- 7 Hz). I am not sure if statistically speaking, the number of data are sufficient to generalized the results. The authors argued that is a common experimental paradigm in fNIRS, however, such protocols are now scarce in the field... Yet, I have to admit that the statistics performed later by the authors seem robust and relevant.

Thank you for your consideration of our statistical analysis as robust and relevant. We are aware that fNIRS devices have lower sampling resolution compared to other devices such as the EEG. However, we would like to highlight that the signal measured by the fNIRS (that is, the BOLD signal extracted from levels of oxyHb) has a cycle of 7-10 seconds. In addition, majority of commercially available fNIRS devices in the market have a similar sampling rate (see Quaresima & Ferrari, 2019 which notes that “transportable/wireless commercially available fNIRS systems have a time resolution of 1 to 10 Hz”) and even if the sampling rate ~7 Hz is “low”, it is still detailed enough to capture the variable that we intend to measure. Furthermore, we agree that fNIRS studies in this specific field are still scarce. However, the number of studies is increasing and we are following a standard procedure with the NIRSport device we used (for example, see Vrana et al, 2016 which used the same NIRSport device with the same signal sampling rate of 7.81Hz as well). Finally, all the data have also been made available online in our data repository – this allows other groups to use our data for further analysis or replication purposes.

3. The authors indicated "multi-distant channel setup" in the section fNIRS recording. To my understanding, the inter-distance probes varied depending on the channels. If yes, the authors have to specify the range of these variations (not just 3cm max as mentioned in the manuscript) and they also need to justify their choice. If the distances are not correct, the fNIRS data cannot take it seriously...

There has been a long debate in the field of fNIRS and EEG on this point. We are using a standard NIRS cap that fits the scalp of the participants. The software that we are using (as with other software available) for the fNIRS data acquisition loads a montage of the channel setup (placement of each emitter/detector) and overcomes this issue by automatically correcting for the interoptode distance based on this pre-loaded montage.

Minor concerns

4. Figure 1 do not correspond to the experimental setup. The authors investigated PFC activation using 20 channels, yet the figure represent a whole-brain fNIRS device with 100 channels.

Thank you for this comment and we have elaborated further in the Figure 1 caption to provide a clearer description of Figure 1 as follows: “Digital rendering of experimental setup depicting (i) 20 channel setup, (ii) NIRS device and (iii) laptop placement.” Figure 1 is a digital rendering of a photo of the actual experimental setup. The cap seen in the figure is a standard NIRS cap where the montage we used was the one described involving 8 emitters and 7 detectors forming the 20 channel setup.

5. Table 1 indicates the correlations between perceptual criticism rating and target relationship. Yet, the authors did not mention the type of correlation at this point of the manuscript.

Thank you for pointing this out - we have included this sentence in the paragraph preceding Table 1 to make it clearer: ” Table 1 indicates the correlations between the PC ratings of the three relationship types.”

6. Figure 3 indicates channel locations. How the authors determined the exact location of the channels? EEG standard, 3D digitizer? The authors need to add the information below the figure.

A standard cap was used where placement of the emitters/detectors on the cap was based on a paired montage in the software which is based on the EEG 10-20 system. We have included this information in the Figure 3 caption as follows: “Montage for probe placement is based on the 10-20 system.”

7. L.358 "replacement of spike artefacts with nearest or random signals". What are the rules to determine such choices?

The software replaces the signal with the nearest signals where possible; otherwise, if there are more adjacent spikes for which replacement with nearest signals is not obvious to determine, it replaces the spike with random signals. However, we would like to stress that participants with significantly “noisy” data were excluded (as noted in the Results section that “Data for one additional male participant were excluded at the pre-processing stage due to poor signal quality.”). Hence, the data of the remaining participants included in the analysis had < 0.01% of spike artefacts.

8. L.438 - 444. The authors cannot interpret the data without any statistics. However, If my understanding is correct, the authors add a similar paragraph with the corresponding stats and figure later L.452 - 459. The authors should thus remove the first paragraph.

We have removed the first paragraph (L438-444).

If the authors can assess all my concerns, the present manuscript should improve our knowledge on the field. Use fNIRS as a methodological approach to investigate perceptual criticism - PFC activation is also interesting.

We hope we have addressed all your concerns. Thank you for your comments that have helped in making our article significantly clearer.
